# *N*′-(3,4-Dimethoxy­benzyl­idene)aceto­hydrazide

**DOI:** 10.1107/S1600536809028608

**Published:** 2009-07-25

**Authors:** Bao-Cheng Zhou, Lu-Ping Lv, Wen-Bo Yu, Wei-Wei Li, Xian-Chao Hu

**Affiliations:** a Department of Applied Chemistry, Zhejiang Sci-tech University, Hangzhou 310018, People’s Republic of China; b Key Laboratory of Advanced Textile Materials and Manufacturing Technology, Ministry of Education, Zhejiang Sci-Tech University, Hangzhou 310018, People’s Republic of China; c Department of Chemical Engineering, Hangzhou Vocational and Technical College, Hangzhou 310018, People’s Republic of China; d Research Center of Analysis and Measurement, Zhejiang University of Technology, Hangzhou 310014, People’s Republic of China

## Abstract

The asymmetric unit of the title compound, C_11_H_14_N_2_O_3_, contains two independent mol­ecules with close conformations; the C=N—N—C torsion angle is 176.4 (1)° in both mol­ecules. In the crystal, inter­molecular N—H⋯O and C—H⋯O hydrogen bonds link the mol­ecules into chains running along the [01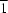] direction.

## Related literature

For general background to the applications of Schiff bases, see: Cimerman *et al.* (1997 ▶); Offe *et al.* (1952 ▶); Richardson *et al.* (1988 ▶). For related structures, see: Li & Jian (2008 ▶); Tamboura *et al.* (2009 ▶).
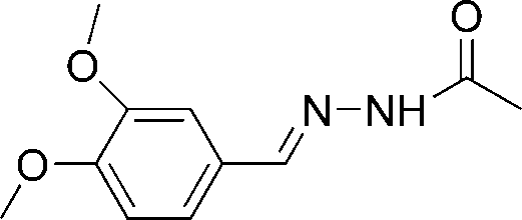


## Experimental

### 

#### Crystal data

C_11_H_14_N_2_O_3_
*M* *_r_* = 222.24
Triclinic, 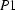

*a* = 8.339 (3) Å
*b* = 8.349 (3) Å
*c* = 8.663 (3) Å
α = 94.717 (12)°
β = 95.210 (8)°
γ = 94.298 (12)°
*V* = 596.6 (3) Å^3^
*Z* = 2
Mo *K*α radiation
μ = 0.09 mm^−1^
*T* = 223 K
0.24 × 0.21 × 0.19 mm

#### Data collection

Bruker SMART CCD area-detector diffractometer
Absorption correction: multi-scan (*SADABS*; Bruker, 2002 ▶) *T*_min_ = 0.987, *T*_max_ = 0.990
3236 measured reflections
2054 independent reflections
1890 reflections with *I* > 2σ(*I*)
*R*_int_ = 0.017

#### Refinement

*R*[*F* ^2^ > 2σ(*F* ^2^)] = 0.036
*wR*(*F* ^2^) = 0.110
*S* = 1.12
2054 reflections
290 parameters
3 restraints
H-atom parameters constrained
Δρ_max_ = 0.18 e Å^−3^
Δρ_min_ = −0.15 e Å^−3^

### 

Data collection: *SMART* (Bruker, 2002 ▶); cell refinement: *SAINT* (Bruker, 2002 ▶); data reduction: *SAINT*; program(s) used to solve structure: *SHELXS97* (Sheldrick, 2008 ▶); program(s) used to refine structure: *SHELXL97* (Sheldrick, 2008 ▶); molecular graphics: *SHELXTL* (Sheldrick, 2008 ▶); software used to prepare material for publication: *SHELXTL*.

## Supplementary Material

Crystal structure: contains datablocks I, global. DOI: 10.1107/S1600536809028608/cv2586sup1.cif

Structure factors: contains datablocks I. DOI: 10.1107/S1600536809028608/cv2586Isup2.hkl

Additional supplementary materials:  crystallographic information; 3D view; checkCIF report

## Figures and Tables

**Table 1:** Hydrogen-bond geometry (Å, °)

*D*—H⋯*A*	*D*—H	H⋯*A*	*D*⋯*A*	*D*—H⋯*A*
N2—H2⋯O4^i^	0.86	2.11	2.950 (3)	165
N2—H2⋯O5^i^	0.86	2.54	3.154 (3)	129
C7—H7⋯O6	0.93	2.52	3.372 (3)	152
C12—H12*B*⋯O3^ii^	0.96	2.51	3.434 (4)	162
C12—H12*C*⋯O6^iii^	0.96	2.45	3.367 (4)	159
C16—H18⋯O2^iv^	0.93	2.45	3.244 (3)	144
N4—H4⋯O3^v^	0.86	2.08	2.907 (3)	161

Symmetry codes: (i) 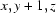; (ii) 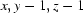; (iii) 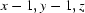; (iv) 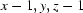; (v) 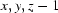.

**Fig. 1.**
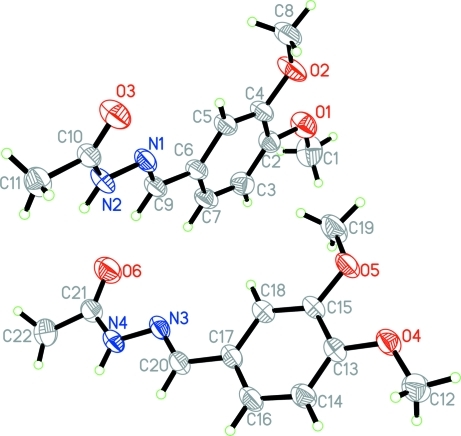
The content of asymmetric unit of the title compound. Displacement ellipsoids are drawn at the 30% probability level.

## supplementary crystallographic information

### Comment

Schiff bases  have attracted much attention due to their possible analytical applications (Cimerman *et al.*, 1997). They are also important ligands, which have been reported to have mild bacteriostatic activity and potential oral iron-chelating drugs for genetic disorders such as thalassemia (Offe *et al.*, 1952; Richardson *et al.*, 1988). Metal complexes based on Schiff bases have received considerable attention because they can be utilized as model compounds of active centres in various complexes (Tamboura *et al.*,  2009). We report here the crystal structure of the title compound.

The title compound (Fig. 1) crystallizes with two independent molecules in the asymmetric unit. The side chains in the two independent molecules have the same orientations, with the C═N—N—C torsion angle being 176.4 (1)° in both molecules. The N1/N2//O3/C9/C10/C11 and N3/N4/O6/C20/C21/C22 planes form dihedral angles of 6.00 (5)° and 4.38 (9)°, respectively, with the C2—C7 and C13—C18 planes. The dihedral angle between the two independent benzene rings is 79.39 (7)°. The bond lengths and angles are comparable to those observed for *N*'-[1-(4-methoxyphenyl)ethylidene]acetohydrazide (Li *et al.*, 2008).

In the crystal structure, the molecules are linked into chains running along the [01-1] by N—H···O and C—H···O hydrogen bonds(Table 1).

### Experimental

3,4-Methoxybenzaldehyde (1.66 g, 0.01 mol) and acetohydrazide (0.74 g, 0.01 mol) were dissolved in stirred methanol (25 ml) and left for 2.5 h at room temperature. The resulting solid was filtered off and recrystallized from ethanol to give the title compound in 85% yield. Single crystals suitable for X-ray analysis were obtained by slow evaporation of an ethanol solution at room temperature (m.p. 468–470 K).

### Refinement

H atoms were positioned geometrically (N-H = 0.86 Å and C-H = 0.93 or 0.96 Å) and refined using a riding model, with *U*_iso_(H) = 1.2*U*_eq_(C,N) and 1.5*U*_eq_(C_methyl_). In the absence of significant anomalous scatterers, 1140 Friedel pairs were averaged.

### Crystal data

**Table:**

C_11_H_14_N_2_O_3_	*Z* = 2
*M**_r_* = 222.24	*F*(000) = 236
Triclinic, *P*1	*D*_x_ = 1.237 Mg m^−^^3^
Hall symbol: P 1	Mo *K*α radiation, λ = 0.71073 Å
*a* = 8.339 (3) Å	Cell parameters from 2054 reflections
*b* = 8.349 (3) Å	θ = 2.4–25.0°
*c* = 8.663 (3) Å	µ = 0.09 mm^−^^1^
α = 94.717 (12)°	*T* = 223 K
β = 95.210 (8)°	Block, colourless
γ = 94.298 (12)°	0.24 × 0.21 × 0.19 mm
*V* = 596.6 (3) Å^3^	

### Data collection

**Table:**

Bruker SMART CCD area-detector diffractometer	2054 independent reflections
Radiation source: fine-focus sealed tube	1890 reflections with *I* > 2σ(*I*)
graphite	*R*_int_ = 0.017
φ and ω scans	θ_max_ = 25.0°, θ_min_ = 2.4°
Absorption correction: multi-scan (*SADABS*; Bruker, 2002)	*h* = −9→9
*T*_min_ = 0.987, *T*_max_ = 0.990	*k* = −9→9
3236 measured reflections	*l* = −10→9

### Refinement

**Table:**

Refinement on *F*^2^	Secondary atom site location: difference Fourier map
Least-squares matrix: full	Hydrogen site location: inferred from neighbouring sites
*R*[*F*^2^ > 2σ(*F*^2^)] = 0.036	H-atom parameters constrained
*wR*(*F*^2^) = 0.110	*w* = 1/[σ^2^(*F*_o_^2^) + (0.0737*P*)^2^ + 0.0315*P*] where *P* = (*F*_o_^2^ + 2*F*_c_^2^)/3
*S* = 1.12	(Δ/σ)_max_ < 0.001
2054 reflections	Δρ_max_ = 0.18 e Å^−^^3^
290 parameters	Δρ_min_ = −0.15 e Å^−^^3^
3 restraints	Extinction correction: *SHELXL97* (Sheldrick, 2008), Fc^*^=kFc[1+0.001xFc^2^λ^3^/sin(2θ)]^-1/4^
Primary atom site location: structure-invariant direct methods	Extinction coefficient: 0.101 (14)

### Special details

**Table:**

Geometry. All esds (except the esd in the dihedral angle between two l.s. planes) are estimated using the full covariance matrix. The cell esds are taken into account individually in the estimation of esds in distances, angles and torsion angles; correlations between esds in cell parameters are only used when they are defined by crystal symmetry. An approximate (isotropic) treatment of cell esds is used for estimating esds involving l.s. planes.
Refinement. Refinement of F^2^ against ALL reflections. The weighted R-factor wR and goodness of fit S are based on F^2^, conventional R-factors R are based on F, with F set to zero for negative F^2^. The threshold expression of F^2^ > 2sigma(F^2^) is used only for calculating R-factors(gt) etc. and is not relevant to the choice of reflections for refinement. R-factors based on F^2^ are statistically about twice as large as those based on F, and R- factors based on ALL data will be even larger.

### Fractional atomic coordinates and isotropic or equivalent isotropic displacement parameters (Å^2^)

**Table:**

	*x*	*y*	*z*	*U*_iso_*/*U*_eq_	
O4	0.0007 (2)	−0.4436 (3)	0.6700 (2)	0.0538 (6)	
O5	0.2416 (3)	−0.2594 (3)	0.8028 (3)	0.0604 (6)	
O2	0.6714 (3)	0.0884 (3)	1.2796 (3)	0.0691 (7)	
O3	−0.0291 (3)	0.4482 (3)	1.2193 (3)	0.0668 (7)	
N2	0.0775 (3)	0.4780 (3)	0.9931 (3)	0.0520 (6)	
H2	0.0686	0.5156	0.9036	0.062*	
O1	0.8549 (3)	0.0361 (3)	1.0637 (3)	0.0659 (7)	
N3	0.2418 (3)	0.2632 (3)	0.5009 (3)	0.0473 (6)	
N1	0.2086 (3)	0.3943 (3)	1.0364 (3)	0.0476 (6)	
C15	0.1580 (3)	−0.1968 (3)	0.6814 (3)	0.0428 (6)	
O6	0.4783 (3)	0.5122 (3)	0.5771 (3)	0.0796 (8)	
C18	0.1933 (3)	−0.0476 (4)	0.6295 (3)	0.0449 (6)	
H17	0.2816	0.0189	0.6767	0.054*	
C6	0.4492 (3)	0.2834 (3)	0.9661 (3)	0.0460 (7)	
N4	0.2566 (3)	0.4016 (3)	0.4235 (3)	0.0508 (6)	
H4	0.1872	0.4136	0.3465	0.061*	
C17	0.0956 (3)	0.0041 (4)	0.5047 (3)	0.0456 (7)	
C2	0.7255 (3)	0.1177 (4)	1.0226 (3)	0.0479 (7)	
C9	0.3061 (4)	0.3708 (4)	0.9334 (3)	0.0474 (7)	
H9	0.2861	0.4097	0.8365	0.057*	
C5	0.4882 (3)	0.2291 (4)	1.1140 (3)	0.0476 (7)	
H6	0.4221	0.2488	1.1932	0.057*	
C20	0.1279 (4)	0.1605 (4)	0.4428 (3)	0.0497 (7)	
H20	0.0619	0.1854	0.3571	0.060*	
C10	−0.0362 (4)	0.5003 (3)	1.0920 (3)	0.0484 (7)	
C14	−0.0720 (4)	−0.2478 (4)	0.4888 (4)	0.0535 (7)	
H14	−0.1612	−0.3137	0.4425	0.064*	
C13	0.0239 (3)	−0.2980 (3)	0.6102 (3)	0.0446 (7)	
C7	0.5492 (4)	0.2542 (4)	0.8501 (3)	0.0508 (7)	
H7	0.5252	0.2903	0.7526	0.061*	
C16	−0.0345 (4)	−0.0966 (4)	0.4350 (4)	0.0556 (8)	
H18	−0.0980	−0.0638	0.3515	0.067*	
C11	−0.1735 (4)	0.5935 (5)	1.0337 (4)	0.0623 (8)	
H11A	−0.1585	0.6212	0.9303	0.093*	
H11B	−0.1758	0.6903	1.1012	0.093*	
H11C	−0.2737	0.5286	1.0324	0.093*	
C4	0.6230 (4)	0.1474 (4)	1.1414 (3)	0.0495 (7)	
C3	0.6859 (4)	0.1710 (4)	0.8788 (4)	0.0548 (8)	
H3	0.7517	0.1510	0.7994	0.066*	
C21	0.3770 (4)	0.5174 (4)	0.4662 (3)	0.0524 (7)	
C12	−0.1272 (4)	−0.5549 (5)	0.5953 (4)	0.0641 (9)	
H12A	−0.1310	−0.6517	0.6478	0.096*	
H12B	−0.1082	−0.5803	0.4887	0.096*	
H12C	−0.2282	−0.5072	0.5992	0.096*	
C22	0.3783 (5)	0.6553 (4)	0.3640 (5)	0.0694 (9)	
H22A	0.2882	0.6378	0.2856	0.104*	
H22B	0.4771	0.6615	0.3151	0.104*	
H22C	0.3704	0.7545	0.4263	0.104*	
C8	0.5745 (5)	0.1116 (5)	1.4029 (4)	0.0672 (9)	
H5A	0.6225	0.0673	1.4932	0.101*	
H5B	0.4687	0.0584	1.3734	0.101*	
H5C	0.5660	0.2248	1.4263	0.101*	
C1	0.9669 (5)	0.0112 (5)	0.9511 (5)	0.0775 (11)	
H1A	1.0525	−0.0478	0.9934	0.116*	
H1B	1.0112	0.1136	0.9244	0.116*	
H1C	0.9125	−0.0489	0.8596	0.116*	
C19	0.3937 (5)	−0.1813 (5)	0.8631 (5)	0.0800 (12)	
H16A	0.4381	−0.2371	0.9475	0.120*	
H16B	0.3805	−0.0720	0.9001	0.120*	
H16C	0.4656	−0.1820	0.7826	0.120*	

### Atomic displacement parameters (Å^2^)

**Table:**

	*U*^11^	*U*^22^	*U*^33^	*U*^12^	*U*^13^	*U*^23^
O4	0.0553 (11)	0.0539 (13)	0.0528 (12)	−0.0019 (9)	−0.0015 (9)	0.0211 (10)
O5	0.0625 (12)	0.0572 (13)	0.0598 (12)	−0.0038 (10)	−0.0176 (10)	0.0294 (10)
O2	0.0710 (15)	0.1024 (19)	0.0436 (11)	0.0453 (14)	0.0073 (10)	0.0281 (12)
O3	0.0708 (15)	0.0814 (16)	0.0526 (13)	0.0172 (12)	0.0043 (11)	0.0239 (12)
N2	0.0547 (13)	0.0591 (15)	0.0458 (14)	0.0141 (11)	−0.0031 (12)	0.0261 (12)
O1	0.0580 (13)	0.0810 (16)	0.0649 (15)	0.0280 (12)	0.0127 (11)	0.0158 (12)
N3	0.0566 (14)	0.0477 (14)	0.0414 (13)	0.0132 (11)	0.0059 (11)	0.0173 (11)
N1	0.0507 (13)	0.0483 (14)	0.0447 (13)	0.0096 (10)	−0.0062 (11)	0.0161 (10)
C15	0.0420 (14)	0.0495 (16)	0.0393 (14)	0.0100 (12)	0.0007 (11)	0.0161 (12)
O6	0.0796 (17)	0.0751 (17)	0.0801 (18)	0.0006 (13)	−0.0203 (14)	0.0187 (14)
C18	0.0451 (14)	0.0492 (16)	0.0418 (14)	0.0081 (12)	0.0012 (11)	0.0125 (12)
C6	0.0512 (15)	0.0472 (16)	0.0384 (14)	0.0000 (12)	−0.0047 (12)	0.0102 (12)
N4	0.0613 (14)	0.0500 (14)	0.0431 (13)	0.0082 (11)	−0.0015 (11)	0.0199 (11)
C17	0.0505 (16)	0.0472 (16)	0.0422 (15)	0.0143 (12)	0.0045 (12)	0.0128 (12)
C2	0.0483 (16)	0.0502 (17)	0.0464 (16)	0.0092 (13)	0.0046 (13)	0.0073 (13)
C9	0.0559 (16)	0.0490 (16)	0.0377 (14)	0.0054 (12)	−0.0041 (12)	0.0151 (12)
C5	0.0508 (16)	0.0535 (17)	0.0398 (14)	0.0120 (13)	0.0009 (12)	0.0089 (12)
C20	0.0583 (17)	0.0485 (17)	0.0444 (15)	0.0142 (13)	−0.0010 (13)	0.0150 (13)
C10	0.0560 (17)	0.0440 (16)	0.0443 (16)	0.0034 (12)	−0.0055 (14)	0.0100 (12)
C14	0.0471 (15)	0.0567 (18)	0.0551 (18)	0.0026 (13)	−0.0091 (13)	0.0126 (14)
C13	0.0463 (15)	0.0473 (16)	0.0425 (15)	0.0068 (12)	0.0039 (12)	0.0154 (12)
C7	0.0595 (18)	0.0554 (17)	0.0379 (14)	0.0002 (14)	0.0021 (13)	0.0138 (13)
C16	0.0579 (17)	0.0571 (19)	0.0532 (18)	0.0137 (14)	−0.0092 (14)	0.0207 (15)
C11	0.063 (2)	0.063 (2)	0.0614 (19)	0.0159 (16)	0.0004 (16)	0.0106 (16)
C4	0.0532 (16)	0.0593 (18)	0.0381 (15)	0.0133 (14)	−0.0005 (12)	0.0137 (13)
C3	0.0588 (17)	0.0587 (18)	0.0487 (17)	0.0019 (14)	0.0106 (14)	0.0117 (14)
C21	0.0583 (18)	0.0516 (18)	0.0488 (17)	0.0116 (14)	0.0021 (14)	0.0097 (13)
C12	0.066 (2)	0.065 (2)	0.060 (2)	−0.0107 (16)	0.0014 (16)	0.0203 (16)
C22	0.085 (2)	0.0533 (19)	0.071 (2)	0.0030 (17)	0.0061 (19)	0.0154 (17)
C8	0.081 (2)	0.087 (2)	0.0413 (17)	0.0353 (19)	0.0107 (16)	0.0201 (16)
C1	0.063 (2)	0.078 (3)	0.099 (3)	0.0206 (18)	0.031 (2)	0.010 (2)
C19	0.073 (2)	0.073 (2)	0.088 (3)	−0.0081 (18)	−0.037 (2)	0.033 (2)

### Geometric parameters (Å, °)

**Table:**

O4—C13	1.367 (3)	C5—H6	0.9300
O4—C12	1.431 (4)	C20—H20	0.9300
O5—C15	1.370 (3)	C10—C11	1.505 (4)
O5—C19	1.417 (4)	C14—C13	1.376 (4)
O2—C4	1.370 (3)	C14—C16	1.405 (4)
O2—C8	1.407 (4)	C14—H14	0.9300
O3—C10	1.216 (4)	C7—C3	1.393 (5)
N2—C10	1.348 (4)	C7—H7	0.9300
N2—N1	1.380 (3)	C16—H18	0.9300
N2—H2	0.8600	C11—H11A	0.9600
O1—C2	1.355 (4)	C11—H11B	0.9600
O1—C1	1.424 (5)	C11—H11C	0.9600
N3—C20	1.270 (4)	C3—H3	0.9300
N3—N4	1.385 (3)	C21—O6	1.225 (4)
N1—C9	1.274 (4)	C21—C22	1.510 (5)
C15—C18	1.379 (4)	C12—H12A	0.9600
C15—C13	1.412 (4)	C12—H12B	0.9600
O6—C21	1.225 (4)	C12—H12C	0.9600
C18—C17	1.410 (4)	C22—H22A	0.9600
C18—H17	0.9300	C22—H22B	0.9600
C6—C7	1.381 (4)	C22—H22C	0.9600
C6—C5	1.413 (4)	C8—H5A	0.9600
C6—C9	1.463 (4)	C8—H5B	0.9600
N4—C21	1.345 (4)	C8—H5C	0.9600
N4—H4	0.8600	C1—H1A	0.9600
C17—C16	1.381 (4)	C1—H1B	0.9600
C17—C20	1.468 (4)	C1—H1C	0.9600
C2—C3	1.377 (4)	C19—H16A	0.9600
C2—C4	1.415 (4)	C19—H16B	0.9600
C9—H9	0.9300	C19—H16C	0.9600
C5—C4	1.370 (4)		
			
C13—O4—C12	117.5 (2)	C17—C16—H18	119.6
C15—O5—C19	118.3 (2)	C14—C16—H18	119.6
C4—O2—C8	117.6 (2)	C10—C11—H11A	109.5
C10—N2—N1	119.6 (2)	C10—C11—H11B	109.5
C10—N2—H2	120.2	H11A—C11—H11B	109.5
N1—N2—H2	120.2	C10—C11—H11C	109.5
C2—O1—C1	117.2 (3)	H11A—C11—H11C	109.5
C20—N3—N4	114.6 (2)	H11B—C11—H11C	109.5
C9—N1—N2	115.8 (2)	C5—C4—O2	125.2 (3)
O5—C15—C18	125.6 (3)	C5—C4—C2	120.5 (2)
O5—C15—C13	114.2 (2)	O2—C4—C2	114.3 (2)
C18—C15—C13	120.2 (2)	C2—C3—C7	121.2 (3)
C15—C18—C17	120.0 (3)	C2—C3—H3	119.4
C15—C18—H17	120.0	C7—C3—H3	119.4
C17—C18—H17	120.0	O6—C21—N4	123.7 (3)
C7—C6—C5	119.2 (3)	O6—C21—N4	123.7 (3)
C7—C6—C9	119.3 (2)	O6—C21—C22	122.4 (3)
C5—C6—C9	121.5 (3)	O6—C21—C22	122.4 (3)
C21—N4—N3	121.4 (2)	N4—C21—C22	114.0 (3)
C21—N4—H4	119.3	O4—C12—H12A	109.5
N3—N4—H4	119.3	O4—C12—H12B	109.5
C16—C17—C18	119.3 (2)	H12A—C12—H12B	109.5
C16—C17—C20	118.0 (2)	O4—C12—H12C	109.5
C18—C17—C20	122.7 (3)	H12A—C12—H12C	109.5
O1—C2—C3	126.3 (3)	H12B—C12—H12C	109.5
O1—C2—C4	115.0 (2)	C21—C22—H22A	109.5
C3—C2—C4	118.6 (3)	C21—C22—H22B	109.5
N1—C9—C6	120.6 (2)	H22A—C22—H22B	109.5
N1—C9—H9	119.7	C21—C22—H22C	109.5
C6—C9—H9	119.7	H22A—C22—H22C	109.5
C4—C5—C6	120.3 (3)	H22B—C22—H22C	109.5
C4—C5—H6	119.8	O2—C8—H5A	109.5
C6—C5—H6	119.8	O2—C8—H5B	109.5
N3—C20—C17	122.9 (3)	H5A—C8—H5B	109.5
N3—C20—H20	118.5	O2—C8—H5C	109.5
C17—C20—H20	118.5	H5A—C8—H5C	109.5
O3—C10—N2	122.5 (3)	H5B—C8—H5C	109.5
O3—C10—C11	122.2 (3)	O1—C1—H1A	109.5
N2—C10—C11	115.3 (3)	O1—C1—H1B	109.5
C13—C14—C16	119.6 (3)	H1A—C1—H1B	109.5
C13—C14—H14	120.2	O1—C1—H1C	109.5
C16—C14—H14	120.2	H1A—C1—H1C	109.5
O4—C13—C14	124.7 (2)	H1B—C1—H1C	109.5
O4—C13—C15	115.3 (2)	O5—C19—H16A	109.5
C14—C13—C15	120.0 (2)	O5—C19—H16B	109.5
C6—C7—C3	120.2 (3)	H16A—C19—H16B	109.5
C6—C7—H7	119.9	O5—C19—H16C	109.5
C3—C7—H7	119.9	H16A—C19—H16C	109.5
C17—C16—C14	120.9 (2)	H16B—C19—H16C	109.5

### Hydrogen-bond geometry (Å, °)

**Table:**

*D*—H···*A*	*D*—H	H···*A*	*D*···*A*	*D*—H···*A*
N2—H2···O4^i^	0.86	2.11	2.950 (3)	165
N2—H2···O5^i^	0.86	2.54	3.154 (3)	129
C7—H7···O6	0.93	2.52	3.372 (3)	152
C12—H12B···O3^ii^	0.96	2.51	3.434 (4)	162
C12—H12C···O6^iii^	0.96	2.45	3.367 (4)	159
C16—H18···O2^iv^	0.93	2.45	3.244 (3)	144
N4—H4···O3^v^	0.86	2.08	2.907 (3)	161

Symmetry codes: (i) *x*, *y*+1, *z*;  (ii) *x*, *y*−1, *z*−1; (iii) *x*−1, *y*−1, *z*; (iv) *x*−1, *y*, *z*−1; (v) *x*, *y*, *z*−1.
